# Adherence-enhancing intervention and relapse in childhood acute lymphoblastic leukemia: results from the Children’s Oncology Group randomized trial ACCL1033

**DOI:** 10.1038/s41375-026-02951-0

**Published:** 2026-04-13

**Authors:** Smita Bhatia, Lindsey Hageman, Yanjun Chen, Aman Wadhwa, F. Lennie Wong, Elizabeth L. McQuaid, David R. Freyer, Nkechi Mba, Paula Aristizabal, Elizabeth Raetz, Wendy Landier

**Affiliations:** 1 Institute for Cancer Outcomes and Survivorship, University of Alabama at Birmingham, Birmingham, AL USA; 2 Department of Population Sciences, City of Hope, Duarte, CA USA; 3 Department of Psychiatry and Human Behavior, Brown University, Providence, RI USA; 4 Cancer and Blood Disease Institute, Division of Hematology/Oncology, Children’s Hospital Los Angeles, Los Angeles, CA USA; 5 Department of Pediatric Hematology/Oncology, Driscoll Children’s Hospital, Corpus Christi, TX USA; 6 Department of Pediatrics, University of California, San Diego, CA USA; 7 Department of Pediatrics, NYU Langone Medical Center, New York, NY USA

**Keywords:** Paediatrics, Translational research

## Introduction

Durable remissions in children with acute lymphoblastic leukemia (ALL) require an 18-to-24-month-long maintenance phase with daily self-administered oral mercaptopurine (6MP) [1–4]. In a previous multicenter study, we showed that 6MP adherence rates <95% were associated with a 2.7-fold higher relapse risk [4]. Over 40% of relapses in children entering maintenance in first remission were attributable to suboptimal 6MP adherence [4–6]. The most common reason for missing 6MP was forgetfulness [5]; adherent patients endorsed parental vigilance to overcome forgetfulness [7].

These findings prompted a multicenter phase III randomized clinical trial (RCT) to test the efficacy of adherence-enhancing strategies in children and adolescents with ALL receiving 6MP during maintenance [8]. Parents of children with ALL who were <12 years old (yo) at enrollment, and parents and adolescents with ALL who were ≥12yo were randomized to receive education alone (EDU) or an intervention package (IP) that addressed facilitators/barriers to adherence (i.e., forgetfulness, parental vigilance) [5, 7]. The primary aim of the trial was to determine the impact of EDU *vs*. IP on 6MP adherence; the results of this trial varied by patient age [8]. While post-intervention 6MP adherence rates were comparably high for EDU *vs*. IP in the <12yo participants (*p* = 0.53), they were significantly higher for IP (*p* = 0.04) among the ≥12yo [8]. In this report we describe results of the exploratory aim that examined relapse risk by intervention arm. We tested the hypothesis that EDU will result in higher relapse risk *vs*. IP among the ≥12yo participants but will have comparable relapse risks among <12yo participants.

## Methods

This investigator-initiated, unblinded, parallel-group, RCT (ACCL1033; NCT01503632) was conducted at 59 Children’s Oncology Group (COG) sites in the US. Enrollment occurred between 2012 and 2018; participants were followed until August 2025. Eligibility criteria (detailed in the primary report) [8] included diagnosis of ALL at age ≤21, in first clinical remission at enrollment in adherence trial, receiving 6MP for maintenance. Therapeutic trials are detailed in Supplementary Table S1. Participants were randomly assigned 1:1 to IP or EDU using stratified block randomization with age at enrollment (<12 y; ≥12 y) and race/ethnicity (non-Hispanic whites, Hispanics, African Americans, others) as stratification factors.

The education program included video vignettes of ALL patients/parents addressing perceived susceptibility/severity of ALL, perceived benefits/barriers to 6MP ingestion, and examples of how patients/parents overcame such barriers. Study participants (EDU and IP) viewed the video in clinic at start of intervention, self-tailoring viewing to fit linguistic preferences (English/Spanish). Intervention components for IP arm included (i) education (as in EDU), (ii) daily personalized text-messages from healthcare provider to parent (of <12yo patient) or patient and parent (of ≥12yo patient) using a HIPAA-compliant web-based application (*MedActionPlan/CT*), and (iii) daily directly supervised therapy by designated parent [8].

For the first 28 days after enrollment, participants in both arms received 6MP from a Medication Event Monitoring system device (MEMS) without intervention to calculate baseline adherence rates. MEMS uses microelectronic technology to record date and time of 6MP bottle openings [9]. Adherence rate was defined as the ratio of days with MEMS openings to days 6MP was prescribed for each patient. Intervention lasted 16 weeks. Participating sites submitted annual reports, detailing dates of last visit, relapse or death for up to 10 y after enrollment.

### Statistical analysis

An intention-to-treat analysis compared relapse risk between intervention arms using sub-distribution hazards regression analysis, treating non-relapse death as competing risk [10]. Analyses were conducted using SAS statistical software v9.4 (SAS Institute).

## Results

Mean (SD) adherence rates were higher among those in complete continuous remission (92%[12%]) vs. those who relapsed (86%[17%], *p* = 0.059). The 5 y cumulative incidence of relapse was higher among non-adherent patients (adherence rate <95%) *vs*. adherent patients (adherence rate ≥95%): 13.4% (95% CI = 6.4–18.4) vs. 6.0% (95% CI = 3.1–8.5), *p* = 0.009 (Supplementary Table S2). Analyses were conducted in the entire cohort by age group (<12 y; ≥12 y). As a *post-hoc* analysis, we examined the risk of relapse in a subgroup of participants with end-of-induction minimal residual disease (EOI MRD) ≤ 1% to minimize the influence of disease biology. EOI MRD status was not available for 71 patients (16%); in such cases, those with favorable cytogenetics were arbitrarily assigned to the EOI MRD ≤ 1% group.

### Entire cohort

#### Age at enrollment < 12 y

**Fig. 1:**
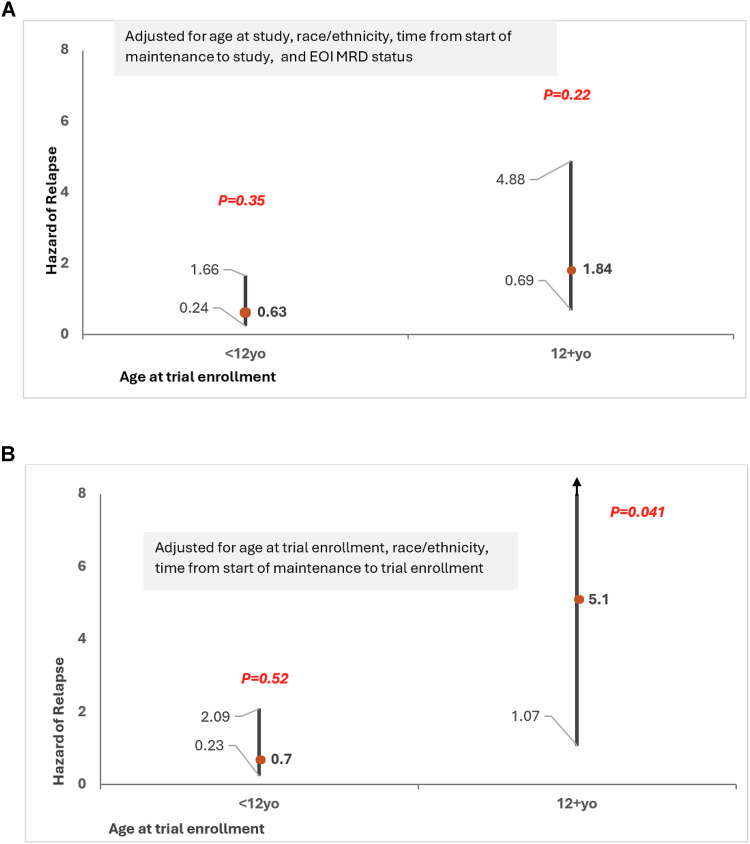
Risk of relapse by intervention arms. **A** Hazard of relapse from start of intervention to date of relapse, death, or last follow-up among those randomized to EDU (reference: IP) in the two age groups (entire cohort). Adjusted for age at trial enrollment, race/ethnicity, time from start of maintenance to study enrollment and EOI MRD. **B** Hazard of relapse from start of intervention to date of relapse, death, or last follow-up among those randomized to EDU (reference: IP) in the two age groups (patients with EOI MRD ≤1%). Adjusted for age at trial enrollment, race/ethnicity, and time from start of maintenance to study enrollment. EDU denotes Education alone; IP denotes the Intervention Package. EOI MRD denotes end of induction MRD.

Baseline characteristics were comparable between participants on EDU (*n* = 143) and IP (*n* = 147) (Table 1). A median follow-up of 7.7 y (0.2–10.3) yielded a 5 y cumulative incidence of relapse of 4.4% *vs*. 7.3% for EDU *vs*. IP, respectively, *p* = 0.31 (Supplementary Table S3). Multivariable analysis adjusted for age at enrollment, race/ethnicity, time from start of maintenance to study enrollment, and EOI MRD revealed no difference in relapse risk between the two arms (EDU: Hazard Ratio [HR] = 0.63, 95% CI = 0.2–1.7, *p* = 0.35; reference: IP) (Fig. 1A, Supplementary Table S4).

**Table 1:** Characteristics of participants by age group and by study arm.

Variables	Entire cohort, Age < 12 y at enrollment (*n* = 290)			Subcohort with EOI MRD ≤ 1%, Age < 12 y at enrollment (*n* = 253)			Entire cohort, Age ≥ 12 y at enrollment (*n* = 154)									Subcohort with EOI MRD ≤ 1%, Age ≥ 12 y at enrollment (*n* = 121)				
	EDU (*n* = 143)	IP (*n* = 147)	*p*-value	EDU (*n* = 124)	IP (*n* = 129)	*p*-value	EDU (*n* = 71)	IP (*n* = 83)							*p*-value	EDU (*n* = 61)	IP (*n* = 60)			*p*-value
Age at ALL diagnosis in years																				
Median (Range)	3.96 (1.2, 10.2)	4.5 (1.0, 10.4)	0.195	3.91 (1.2, 10.2)	4.63 (1.0, 10.4)	0.077	14.48 (9.8, 19.8)	14.09 (10.1, 20.6)							0.452	14.48 (9.8, 19.8)	13.84 (10.5,20.6)			0.246
Age at adherence trial enrollment in years																				
Median (Range)	5.63 (2.6, 11.6)	6.12 (2.4, 11.9)	0.169	5.58 (2.6, 11.5)	6.42 (2.4, 11.9)	0.065	16.42 (12.1, 21.6)	15.7(12.4, 22.3)							0.494	16.42 (12.1, 21.6)	15.52 (12.4,22.3)			0.237
Time from start of maintenance to study participation in years																				
Median (range)	0.9 (0.46, 2.09)	0.91 (0.46, 2.24)	0.511	0.89 (0.46, 2.09)	0.9 (0.46, 2.24)	0.594	0.88 (0.46, 2.05)	0.9(0.48, 2.09)							0.439	0.92 (0.46, 1.93)	0.86 (0.48,2.05)			0.859
Average 6MP dose intensity																				
Median (range)	0.89 (0.3, 1.59)	0.89 (0.38, 2)	0.672	0.89 (0.3, 1.59)	0.89 (0.38, 2)	0.881	0.93 (0.3, 1.89)	0.85 (0.24, 1.68)							0.692	0.91 (0.3, 1.89)	0.83 (0.24,1.68)			0.669
Average methotrexate dose intensity																				
Median (range)	0.83 (0.36, 2.41)	0.87 (0.35, 3.21)	0.365	0.88 (0.36, 2.41)	0.88 (0.35, 2.22)	0.727	0.9 (0.33, 1.68)	0.81 (0.43, 3.74)							0.578	0.89 (0.35, 1.68)	0.78 (0.43,3.74)			0.548
Sex, *n* (%)																				
Female	50 (34.97%)	49 (33.33%)	0.770	43 (34.68%)	44 (34.11%)	0.924	16 (22.54%)	27 (32.53%)							0.168	14 (22.95%)	20 (33.33%)			0.204
Male	93 (65.03%)	98 (66.67%)		81 (65.32%)	85 (65.89%)		55 (77.46%)	56 (67.47%)								47 (77.05%)	40 (66.67%)			
Race/Ethnicity, *n* (%)																				
Other	18 (12.59%)	22 (14.97%)	0.872	16 (12.9%)	20 (15.5%)	0.837	5 (7.04%)	6 (7.23%)							0.895	5 (8.2%)	6 (10%)			0.877
Black	16 (11.19%)	13 (8.84%)		13 (10.48%)	10 (7.75%)		7 (9.86%)	7 (8.43%)								6 (9.84%)	5 (8.33%)			
Hispanic	49 (34.27%)	51 (34.69%)		41 (33.06%)	44 (34.11%)		30 (42.25%)	40 (48.19%)								24 (39.34%)	27 (45%)			
White	60 (41.96%)	61 (41.5%)		54 (43.55%)	55 (42.64%)		29 (40.85%)	30 (36.14%)								26 (42.62%)	22 (36.67%)			
Paternal education, *n* (%)																				
<High school	50 (34.97%)	67 (45.58%)	0.088	44 (35.48%)	56 (43.41%)	0.234	32 (45.07%)	46 (55.42%)							0.212	28 (45.9%)	35 (58.33%)			0.313
≥High school	85 (59.44%)	75 (51.02%)		73 (58.87%)	68 (52.71%)		32 (45.07%)	30 (36.14%)								27 (44.26%)	23 (38.33%)			
Missing	8 (5.59%)	5 (3.4%)		7 (5.65%)	5 (3.88%)		7 (9.86%)	7 (8.43%)								6 (9.84%)	2 (3.33%)			
Maternal education, *n* (%)																				
<High school	40 (27.97%)	51 (34.69%)	0.202	36 (29.03%)	44 (34.11%)	0.318	26 (36.62%)	34 (40.96%)							0.599	21 (34.43%)	22 (36.67%)			0.848
≥High school	101 (70.63%)	93 (63.27%)		88 (70.97%)	82 (63.57%)		42 (59.15%)	46 (55.42%)								38 (62.3%)	37 (61.67%)			
Missing	2 (1.4%)	3 (2.04%)		0(0%)	3 (2.33%)		3 (4.23%)	3 (3.61%)								2 (3.28%)	1 (1.67%)			
Annual household income, *n* (%)																				
<$20,000	33 (23.08%)	36 (24.49%)	0.354	27 (21.77%)	30 (23.26%)	0.718	20 (28.17%)	23 (27.71%)							0.068	17 (27.87%)	17 (28.33%)			0.077
$20,000–$50,000	31 (21.68%)	41 (27.89%)		28 (22.58%)	34 (26.36%)		23 (32.39%)	18 (21.69%)								20 (32.79%)	12 (20%)			
>$50,000	70 (48.95%)	61 (41.5%)		60 (48.39%)	57 (44.19%)		18 (25.35%)	37 (44.58%)								16 (26.23%)	28 (46.67%)			
Missing	9 (6.29%)	9 (6.12%)		9 (7.26%)	8 (6.2%)		10 (14.08%)	5 (6.02%)								8 (13.11%)	3 (5%)			
Blast cytogenetics, *n* (%)																				
Normal	57 (39.86%)	57 (38.78%)	0.880	43 (34.68%)	44 (34.11%)	0.989	50 (70.42%)	53 (63.86%)							0.124	42 (68.85%)	36 (60%)			0.092
Favorable^a^	74 (51.75%)	75 (51.02%)		71 (57.26%)	75 (58.14%)		7 (9.86%)	18 (21.69%)								7 (11.48%)	16 (26.67%)			
Unfavorable^b^	12 (8.39%)	15 (10.2%)		10 (8.06%)	10 (7.75%)		14 (19.72%)	12 (14.46%)								12 (19.67%)	8 (13.33%)			
Minimal residual disease at end of induction																				
≤1%	124 (86.71%)	129 (87.76%)	0.7903				61 (85.92%)	60 (72.29%)							0.0399					
>1%	19 (13.29%)	18 (12.24%)					10 (14.08%)	23 (27.71%)												
Baseline adherence																				
Median (range)	100% (32–100)	97% (36–100)	0.261	100% (32, 100)	100% (36, 100)	0.468	96% (46–100)	96% (29–100)							0.749	97% (46, 100)	96% (29, 100)			0.896
Length of follow-up in years																				
Median (range)	7.75 (0.85–9.45)	7.55 (0.21–10.27)	0.234	8.15 (0.93, 10.97)	7.94 (0.21, 10.27)	0.231	4.86 (0.08–8.53)	4.79 (0.15–9.45)							0.921	5.45 (0.15,9. 41)	5.74 (0.2, 9.5)			0.566
Relapse																				
Yes	7 (4.90%)	11 (7.48%)	0.361	5 (4.03%)	8 (6.2%)	0.435	10 (14.08%)	7 (8.43%)							0.265	8 (13.11%)	2 (3.33%)			0.051

ALL denotes acute lymphoblastic leukemia; 6MP denotes mercaptopurine; EDU denotes the education arm; IP denotes the intervention package arm.

^a^Favorable cytogenetics included one or more of the following: t(12;21), hyperdiploidy, trisomy 4 and 10, or trisomy 4, 10 and 17.

^b^Unfavorable cytogenetics included one or more of the following: t(9;22), t(4;11), hypodiploidy, or extreme hypodiploidy.

#### Age at enrollment ≥12 y

Baseline characteristics were comparable between participants on EDU (*n* = 71) and IP (*n* = 83) (Table 1), except for a lower prevalence of patients with EOI MRD ≤ 1% on EDU *vs*. IP arms (14% *vs*. 28%, *p* = 0.04). Median follow-up of 4.86 y (0.08–9.45) yielded a 5 y cumulative incidence of relapse of 15.1% *vs*. 10.5% for EDU *vs*. IP, respectively, *p* = 0.47 (Supplementary Table S3). Multivariable analysis adjusted for factors listed above revealed a higher relapse risk among patients on EDU (HR = 1.84, 95% CI = 0.7–4.9, *p* = 0.22; reference: IP arm) (Fig. 1A, Supplementary Table S4); however, this difference did not reach statistical significance.

### Sub-cohort with MRD ≤1%

#### Age at enrollment <12 y

Baseline characteristics of participants on EDU (*n* = 124) and IP (*n* = 129) (Table 1) were comparable. A median follow-up of 8.1 y (0.2–11.0) yielded a 5 y cumulative incidence of relapse for EDU *vs*. IP was 3.5% *vs*. 5.3%, *p* = 0.48 (Supplementary Table S3). Multivariable analysis adjusted for age at study, race/ethnicity, and time from start of maintenance to enrollment revealed no difference in relapse risk between the two arms (EDU: HR = 0.7, 95% CI = 0.2–2.1; *p* = 0.52; reference: IP arm) (Fig. 1B, Supplementary Table S5).

#### Age at enrollment ≥12 y

Baseline characteristics were comparable between participants on EDU (*n* = 61) and IP (*n* = 60) (Table 1). A median follow-up of 5.5 y (0.2–9.5) yielded a 5 y cumulative incidence of relapse of 14.8% vs. 3.9% for EDU *vs*. IP, respectively, *p* = 0.07 (Supplementary Table S3). Multivariable analysis revealed a higher risk of relapse among patients on the EDU arm (HR = 5.1, 95% CI = 1.1–24.4, *p* = 0.04; reference: IP arm) (Fig. 1B, Supplementary Table S5).

## Discussion

We have previously reported the primary results of our multicenter RCT [8], showing that the adherence-enhancing intervention resulted in higher adherence in ≥12yo patients with ALL, but comparably high adherence rates for both arms among the <12yo. Adolescents are especially likely to have suboptimal adherence [5, 6], because of increasing assumption of independence and decreasing parental supervision, resulting in this subgroup drawing benefit from our adherence-enhancing intervention [8]. In the current report, we describe whether the benefit from improving 6MP adherence resulted in reduction in relapse risk, testing the hypothesis that the hazard of relapse would be comparable between the two arms in the younger age group, but would be higher in the older age group randomized to EDU *vs*. IP. We tested this hypothesis in the entire cohort and also among those with EOI MRD ≤ 1% to ensure that we were examining the impact of the intervention on relapses likely attributable to poor adherence and not to disease biology.

Among <12yo participants, we observed no difference in relapse risk between the two arms in the entire cohort, as well as among those with end of induction MRD was ≤1%. However, among ≥12yo participants, we observed a two-fold greater hazard of relapse in the entire cohort, and a 5-fold greater hazard of relapse in the sub-cohort with EOI MRD ≤ 1% in the EDU vs. IP arm. These findings suggest that improving 6MP adherence in the older age group may have helped prevent disease relapse.

Findings of this study should be interpreted in the context of certain limitations. In a *post hoc* analysis, we excluded patients with EOI MRD > 1% to focus on relapses that would be more likely to be attributable to non-adherence to therapy rather than disease biology. EOI MRD (a measure of clone-specific leukemic markers) is a strong prognostic marker and is highly predictive of relapse in ALL. EOI MRD cut points employed to make clinical decisions include <0.01% (most favorable), 0.01% to <1.0% (intermediate), and ≥1.0% (unfavorable) [11]. Our results were comparable when patients with the subcohort were restricted to EOI MRD < 0.01% (Supplementary Table S6). Furthermore, in the cohort with EOI MRD ≤ 1%, there was no difference in other high-risk features between those in remission *vs*. those who had relapsed (Supplementary Table S7). The relatively small number of relapse events as well as limitations associated with any *post hoc* analyses need to be acknowledged. These limitations notwithstanding, to our knowledge, this study is the first RCT in pediatric oncology that examined the impact of a 6MP adherence-enhancing intervention on relapse risk, showing that improvement in adherence may have reduced relapse risk. These findings support continued focus on adherence-enhancing strategies in patients at risk for poor adherence. Along these lines, we are currently conducting two parallel RCTs tailored to baseline adherence, with intensification of the intervention for the baseline non-adherers and a parent/patient-empowered intervention for baseline adherers.

## Supplementary information

Supplementary Material

## Data Availability

The datasets generated and/or analyzed during the current study are not publicly available due to restrictions related to participant and protocol-wide study permissions, but are available from the corresponding author on reasonable request and with permission from the Children’s Oncology Group.
