# Supplementary material for: Adherence-enhancing intervention and relapse in childhood acute lymphoblastic leukemia: results from the Children’s Oncology Group randomized trial ACCL1033
Source: Leukemia. 2026 Apr 13;40(5):1062–6. doi: 10.1038/s41375-026-02951-0 (PMC13149298; doi:10.1038/s41375-026-02951-0)
Supplement: Supplementary file 1 — Supplementary Material [file 41375_2026_2951_MOESM1_ESM.docx]

**Supplemental Material**

**Bhatia et al.**

**Table S1. Summary of Therapeutic Trials used for Adherence trial Participants**

| **Trial** | **Targeted Group**  **and Enrollment years** | **Randomization(s)** | **Survival** | **Key Findings** |
| --- | --- | --- | --- | --- |
| **COG-AALL0932**  **NCT1190930** | Standard Risk B-ALL  2010-2018 | Oral methotrexate during maintenance: 20 vs. 40 mg/m^2^ weekly  Vincristine and dexamethasone pulses during maintenance: 4 vs 12 wks | 5y DFS: 92.0% (91.1%-92.8%)  5y OS: 96.8% (96.2%-97.3) | No advantage for 20 vs. 40 mg/m^2^ of weekly oral methotrexate  Equivalent outcomes for 4 vs. 12 week vincristine and dexamethasone pulses  Established every 12 week vincristine/ dexamethasone pulses and 20 mg/m2 of weekly oral methotrexate as standard of care. |
| **AALL1131**  **NCT02883049** | High Risk B-ALL  2012-2018 | Etoposide/CPM/Clofarabine postinduction in very high risk patients  IT methotrexate vs. triple intrathecal therapy with methotrexate, hydrocortisone and cytarabine as CNS prophylaxis | 5y postinduction DFS (±SE) and OS (±SE): 93.2% ±2.1% v 90.6% ±2.3% (P=.85) and 96.3% ±1.5% v 96.7% ±1.4% (P=.77), for those randomized between IT methotrexate and triple IT therapy, respectively | Etoposide/CPM/Clofarabine arm closed early due to toxicity  intrathecal triple therapy did not improve outcomes or change patterns of relapse.  The standard of care for CNS prophylaxis for children with B-ALL and no overt CNS involvement remains IT MTX. |
| **AALLL0434**  **NCT00408005** | T-ALL  2007-2012 | 2 x 2 randomization comparing Capizzi-style intravenous methotrexate vs. high-dose IV methotrexate, with or without Nelarabine, during the 8-week interim maintenance phase | 5y EFS and OS for patients with T-ALL: 83.8% (95%CI, 81.2-86.4%) and 89.5% (95%CI, 87.4-91.7%), respectively. | A significant advantage for Capizzi methotrexate in interim maintenance was observed with 5-year DFS (*p* = .005) and OS (*p*=.04) rates of 91.5% (95% CI, 88.1–94.8%) and 93.7% (95% CI, 90.8–96.6%) compared with 85.3% (95% CI, 81.0–89.5%) and 89.4% (95% CI, 85.7–93.2%) for high-dose methotrexate.  Additionally, there was a DFS advantage for nelarabine, largely attributable to reduction in CNS relapses. |
| **AALL0232**  **NCT00075725** | High Risk B-ALL  2004-2011 | 2 x 2 factorial design comparing dexamethasone (14 days) vs. prednisone (28 days) during induction, and high-dose methotrexate vs Capizzi escalating-dose methotrexate plus pegaspargase during interim maintenance 1. | 5y EFS 82% vs. 75.4% (P=.006) favoring high-dose methotrexate; mature final data showed 5y EFS 79.6% for high-dose methotrexate vs. 75.2% for Capizzi methotrexate (P=.008) | High-dose methotrexate decreased both marrow and CNS recurrences  Dexamethasone given during induction was of benefit to younger children (1-9y) but was of no benefit and was associated with a higher risk of osteonecrosis among children 10y and older. |
| **AALL1231**  **NCT02112916** | T-ALL and T-LL  2014-2017 | Participants were randomly assigned to a modified augmented Berlin-Frankfurt-Munster chemotherapy regimen with/without bortezomib during induction and delayed intensification. | 4y EFS and OS were 81.9% ±1.5% vs. 87.0% ± 1.3%, respectively | Bortezomib did not improve EFS/OS in T-ALL.  High-dose methotrexate was used to improve CNS control  Cranial radiation was used in <10% of patients and the reduction in prophylactic cranial radiation therapy was successful. |
| **AALL0331**  **NCT00103285** | Standard Risk B-ALL  2005-2010 | Following a 3-drug induction, patients meeting low-risk criteria (no extramedullary disease, <5% marrow blasts by day 15, end-induction MD <0.1%, and favorable cytogenetics [ETV6-RUNX1 fusion or triple trisomy of chromosomes 4, 10, and 17]) were randomized to standard COG low-intensity therapy with or without four additional pegaspargase doses at 3-week intervals during consolidation and interim maintenance. | 6y continuous complete remission (CCR) and OS for the entire cohort were 94.7% ± 0.6% and 98.7% ± 0.3%, respectively.  CCR rates were similar between arms, with no difference in OS. | Standard COG therapy without intensified pegaspargase cures nearly all children with B-ALL identified as low-risk by clinical, early response, and favorable cytogenetic criteria. |

**Table S2. Cumulative incidence of relapse among adherers and non-adherers in the entire cohort**

| **Years since study entry** | **Non-adherers**  **Mean adherence <95%** | **Adherers**  **Mean adherence ≥95%** | **p-value** |
| --- | --- | --- | --- |
| **2 years** | 7.4% (2.5%-11.5%) | 3.4% (1.3%-5.3%) | **0.0085** |
| **5 years** | 13.4% (6.4%-18.4%) | 6% (3.1%-8.5%) |  |

**Table S3. Cumulative incidence of relapse and adjusted hazard of relapse by study arm in the two age groups – overall, and by MRD categories**

|  | **<12 years** | | **≥12 years** | |
| --- | --- | --- | --- | --- |
|  | **Cumulative Incidence of relapse at 5y** | **p-value** | **Cumulative Incidence of relapse at 5y** | **p-value** |
| **Entire Cohort** | | | | |
| EDU | 4.4% | 0.31 | 15.1% | 0.47 |
| IP | 7.3% |  | 10.5% |  |
| **MRD ≤1%** | | | | |
| EDU | 3.5% | 0.48 | 14.8% | 0.07 |
| IP | 5.3% |  | 3.9% |  |

MRD denotes minimal residual disease; IP denotes Intervention Package arm; EDU denotes Education alone arm

**Supplemental Table 4.** **Hazard of relapse by intervention arm in the two age groups, in the entire cohort**

|  | **Age <12y at study participation** | | **Age ≥12y at study participation** | |
| --- | --- | --- | --- | --- |
| **Variables** | **HR (95% CI)** | **p-value** | **HR (95% CI)** | **p-value** |
| **Study arm** | | | | |
| Edu vs. IP | 0.63 (0.24,1.66) | 0.354 | 1.84 (0.69,4.88) | 0.220 |
| **Age at Study** | | | | |
| Per year increase | 1.24 (1.02,1.51) | 0.029 | 1.11 (0.91,1.36) | 0.308 |
| **Race/Ethnicity (reference: non-Hispanic White)** | | | | |
| Other vs. NHW | 1.06 (0.39,2.87) | 0.911 | 1.44 (0.48,4.27) | 0.516 |
| **Time from start of maintenance to study enrollment** | | | | |
| Per year increase | 1.02 (0.32,3.25) | 0.968 | 0.29 (0.09,0.99) | 0.048 |
| **Minimal Residual Disease (reference: MRD >1%)** | | | | |
| ≤1% | 0.33 (0.11,0.95) | 0.040 | 0.29 (0.1,0.84) | 0.023 |

EDU denotes Education alone arm; IP denotes the Intervention Package arm. HR denotes Hazard Ratio; CI denotes Confidence Interval; NHW denotes Non-Hispanic White

**Table S5.** **Hazard of relapse by intervention arm in the two age groups, among patients with end-of-induction MRD ≤1%**

|  | **Age <12y at study participation** | | **Age ≥12y at study participation** | |
| --- | --- | --- | --- | --- |
| **Variables** | **HR (95% CI)** | **p-value** | **HR (95% CI)** | **p-value** |
| **Study arm (reference: IP)** | | | | |
| EDU vs. IP | 0.7 (0.23,2.09) | 0.519 | 5.1 (1.07,24.37) | 0.041 |
| **Age at Study** | | | | |
| Per year increase | 1.29 (1.04,1.61) | 0.022 | 1.29 (1.03,1.62) | 0.027 |
| **Race/Ethnicity (reference: non-Hispanic White)** | | | | |
| Other vs. NHW | 1.2 (0.39,3.75) | 0.751 | 3.82 (0.77,19.01) | 0.102 |
| **Time from start of maintenance to study enrollment** | | | | |
| Per year increase | 0.94 (0.26,3.45) | 0.930 | 0.17 (0.03,1.01) | 0.051 |

EDU denotes Education alone arm; IP denotes the Intervention Package arm. HR denotes Hazard Ratio; CI denotes Confidence Interval; NHW denotes Non-Hispanic White

**Table S6.** Hazard of relapse by intervention arm – comparison of cohorts with different MRD cut points

|  | **Multivariable regression analysis* including EOI MRD ≤1%** | | | **Multivariable regression analysis* including EOI MRD <0.01%** | | | |
| --- | --- | --- | --- | --- | --- | --- | --- |
|  | **HR (95%CI)** | **p-value** | | **HR (95%CI)** | | **p-value** | |
| **Age at study ≥12y** | | | | | | | |
| EDU vs. IP | 5.1 (1.07,24.37) | | 0.041 | | 6.79 (0.64,72.56) | | 0.11 |
| **Age at study <12y** | | | | | | | |
| EDU vs. IP | 0.7 (0.23,2.09) | | 0.52 | | 0.24 (0.03,2) | | 0.19 |

*Adjusted for age at study, race/ethnicity, time from start of maintenance to study enrollment

**Table S7.** Prognostic factors by remission status among ≥12yo with end-of-induction MRD ≤1% (n=121)

|  | **EDU (n=61)** | | | **IP (n=60)** | | |
| --- | --- | --- | --- | --- | --- | --- |
|  | **Relapse (n=8)** | **Remission (n=53)** | **p-value** | **Relapse (n=2)** | **Remission (n=58)** | **p-value** |
| **WBC at diagnosis** | | | | | | |
| Mean (range) | 106.1  (2.8-396) | 41.3  (0.6-590) | 0.56 | 31.0 (2.3,59.7) | 59.2 (0.52,637.08) | 0.9836 |
| **Blast cytogenetics** | | | | | | |
| Normal | 4 (50%) | 38 (71.7%) | 0.29 | 1 (50%) | 35 (60.34%) | 0.32 |
| Favorable* | 1 (12.5%) | 6 (11.32%) |  | 0(0%) | 16 (27.59%) |  |
| Unfavorable** | 3 (37.5%) | 9 (16.98%) |  | 1 (50%) | 7 (12.07%) |  |

*Favorable cytogenetics included one or more of the following: t(12;21), hyperdiploidy, trisomy 4 and 10, or trisomy 4, 10 and 17.

**Unfavorable cytogenetics included one or more of the following: t(9;22), t(4;11), hypodiploidy, or extreme hypodiploidy.

**References**

1. Angiolillo AL, Schore RJ, Kairalla JA, et al: Excellent Outcomes With Reduced Frequency of Vincristine and Dexamethasone Pulses in Standard-Risk B-Lymphoblastic Leukemia: Results From Children's Oncology Group AALL0932. J Clin Oncol 39:1437–1447, 2021

2. Salzer WL, Burke MJ, Devidas M, et al: Impact of Intrathecal Triple Therapy Versus Intrathecal Methotrexate on Disease-Free Survival for High-Risk B-Lymphoblastic Leukemia: Children's Oncology Group Study AALL1131. J Clin Oncol 38:2628–2638, 2020

3. Winter SS, Dunsmore KP, Devidas M, et al: Improved Survival for Children and Young Adults With T-Lineage Acute Lymphoblastic Leukemia: Results From the Children's Oncology Group AALL0434 Methotrexate Randomization. J Clin Oncol 36:2926–2934, 2018

4. Larsen EC, Devidas M, Chen S, et al: Dexamethasone and High-Dose Methotrexate Improve Outcome for Children and Young Adults With High-Risk B-Acute Lymphoblastic Leukemia: A Report From Children's Oncology Group Study AALL0232. J Clin Oncol 34:2380–8, 2016

5. Teachey DT, Devidas M, Wood BL, et al: Children's Oncology Group Trial AALL1231: A Phase III Clinical Trial Testing Bortezomib in Newly Diagnosed T-Cell Acute Lymphoblastic Leukemia and Lymphoma. J Clin Oncol 40:2106–2118, 2022

6. Mattano LA, Jr., Devidas M, Maloney KW, et al: Favorable Trisomies and ETV6-RUNX1 Predict Cure in Low-Risk B-Cell Acute Lymphoblastic Leukemia: Results From Children's Oncology Group Trial AALL0331. J Clin Oncol 39:1540–1552, 2021
